# Household Exposure to Paint and Petroleum Solvents, Chromosomal Translocations, and the Risk of Childhood Leukemia

**DOI:** 10.1289/ehp.11927

**Published:** 2008-10-10

**Authors:** Ghislaine Scélo, Catherine Metayer, Luoping Zhang, Joseph L. Wiemels, Melinda C. Aldrich, Steve Selvin, Stacy Month, Martyn T. Smith, Patricia A. Buffler

**Affiliations:** 1 School of Public Health, University of California–Berkeley, Berkeley, California, USA;; 2 Laboratory of Molecular Epidemiology and; 3 Department of Medicine, University of California–San Francisco, San Francisco, California, USA;; 4 Kaiser Permanente, Oakland, California, USA

**Keywords:** case-control study, children, leukemia, paint, petroleum, solvents

## Abstract

**Background:**

Few studies have examined the association between home use of solvents and paint and the risk of childhood leukemia.

**Objectives:**

In this case–control study, we examined whether the use of paint and petroleum solvents at home before birth and in early childhood influenced the risk of leukemia in children.

**Methods:**

We based our analyses on 550 cases of acute lymphoblastic leukemia (ALL), 100 cases of acute myeloid leukemia (AML), and one or two controls per case individually matched for sex, age, Hispanic status, and race. We conducted further analyses by cytogenetic subtype. We used conditional logistic regression techniques to adjust for income.

**Results:**

ALL risk was significantly associated with paint exposure [odds ratio (OR) = 1.65; 95% confidence interval (CI), 1.26–2.15], with a higher risk observed when paint was used postnatally, by a person other than the mother, or frequently. The association was restricted to leukemia with translocations between chromosomes 12 and 21 (OR = 4.16; 95% CI, 1.66–10.4). We found no significant association between solvent use and ALL risk overall (OR = 1.15; 95% CI, 0.87–1.51) or for various cytogenetic subtypes, but we observed a significant association in the 2.0- to 5.9-year age group (OR = 1.55; 95% CI, 1.07–2.25). In contrast, a significant increased risk for AML was associated with solvent (OR = 2.54; 95% CI, 1.19–5.42) but not with paint exposure (OR = 0.64; 95% CI, 0.32–1.25).

**Conclusions:**

The association of ALL risk with paint exposure was strong, consistent with a causal relationship, but further studies are needed to confirm the association of ALL and AML risk with solvent exposure.

Leukemia is the most common cancer in children worldwide (0–14 years of age), with more than 2,600 cases diagnosed annually in the United States (Ferlay et al. 2004; Parkin et al. 2005). The main two histological groups are acute lymphoblastic leukemia (ALL) and acute myeloid leukemia (AML), representing 80% and 13% of all cases in the United States, respectively (Parkin et al. 2005). Recently, greater heterogeneity of childhood leukemias has been recognized, and they have been subclassified according to their cytogenetics. The causes of childhood leukemia remain largely unknown, and recognized risk factors, such as genetic conditions (e.g., Down syndrome), ionizing radiation, and chemotherapeutic agents, explain only a small proportion of cases (Buffler et al. 2005).

Exposures to paint and solvents have been suggested as potential risk factors of childhood leukemia. Paint is a generic name for a number of different products, and its potential toxicity depends on the types of pigments, resins, and solvents used in its manufacture (Kirk-Othmer 2006). One of the two major groups of paints is latex paints, for which the resin is acrylic-, vinyl-, or styrene-based and the solvent is water, with the customary addition of glycol ethers and coalescent aid that helps the resins flow together, aiding in film formation. The other is the alkyd paints or oil-based resin paints, in which the solvent is usually petroleum-based and organic, such as toluene or xylene. Similarly, petroleum solvents encompass a wide variety of materials derived from crude oil [International Agency for Research on Cancer (IARC) Monograph Working Group 1989], including many that are widely used in the home, such as paint thinner, spot remover, gasoline, kerosene, and lubricating oil.

Most previous epidemiologic studies have examined the impact of paint and solvents on the risk of childhood leukemia through parental occupational exposures and have reported conflicting results (Colt and Blair 1998; Feychting et al. 2001; Infante-Rivard et al. 2005; McKinney et al. 2003; Schüz et al. 2000; Shu et al. 1999). Only four studies described results for home use of these products (Alderton et al. 2006; Freedman et al. 2001; Infante-Rivard et al. 2005; Lowengart et al. 1987), with two reporting a suggestive association with paint exposure (Freedman et al. 2001; Lowengart et al. 1987). Inconsistencies among the previous findings may be attributable to heterogeneous time windows of exposures, multiple sources of exposures, and heterogeneous types of leukemia analyzed together.

Our objective was to determine whether an association exists between the risk of childhood leukemia and household exposure to paint and/or petroleum solvents, during different time windows of exposure (preconception, pregnancy, and early childhood), for ALL and AML separately, and by major cytogenetic subtype. We based analyses on data from the Northern California Childhood Leukemia Study (NCCLS), a large population-based case–control study that covers 35 California counties.

## Material and Methods

The study was approved by the University of California Committee for the Protection of Human Subjects, the California Health and Human Services Agency Committee for the Protection of Human Subjects, and the institutional review boards of the nine participating hospitals. We obtained written informed consent from the parents of all participating subjects before interviews.

### Study population

The NCCLS is an ongoing, population-based case–control study that began in 1995. The present analysis used data from two phases: cases diagnosed from 31 August 1995 to 30 November 1999 in 17 counties in the San Francisco Bay Area (phase 1), and cases diagnosed from 1 December 1999 to 6 December 2005 in the same area and an additional 18 counties in California Central Valley (phase 2). We identified incident cases on the basis of *International Classification of Diseases for Oncology* (Fritz et al. 2000; Percy et al. 1990) using rapid case ascertainment procedure in four (phase 1) to nine (phase 2) pediatric hospitals, usually within 72 hr after diagnosis. Cases were eligible if they were < 15 years of age at diagnosis, had no prior cancer diagnosis, lived in the study area, and their biological parents spoke either English or Spanish. Comparison of case ascertainment in the 35-county study area to the California Cancer Registry data (1997–2003; http://www.ccrcal.org/) showed that the NCCLS ascertained 96% of children diagnosed with leukemia in the four phase 1 participating hospitals, and 93% in the nine phase 2 hospitals. Overall, the cases diagnosed at the participating hospitals and ascertained through the NCCLS protocol represent approximately 76% of the diagnosed cases in the 35 study counties. Of the eligible cases, 86% consented to participate.

We randomly selected one or two control children who resided within the study area from birth certificates supplied by the California Department of Health Services (Sacramento, CA) and individually matched them to cases on date of birth, sex, Hispanic ethnicity (we considered a child Hispanic if either parent was Hispanic), and maternal race. Contact failed for 13% selected controls; we then randomly selected another matched control. Of the controls that we located, the participation rate, defined as the number of participating controls divided by the number of eligible or presumed eligible controls, was 68%. Further description of NCCLS control recruitment has been previously published (Kwan et al. 2007; Ma et al. 2004).

Since 1995, data for 669 case–control sets have been accumulated, with a total of 1,558 children. For the present analyses, we included only ALL and AML cases. In addition, we excluded case–control sets with missing data for exposure to paint or solvent for cases or controls from the analyses, resulting in 650 case–control sets (438 pairs and 212 triplets) with 550 case–control sets for ALL analyses and 100 case–control sets for AML. Table 1 presents selected characteristics of the participating children.

### Cytogenetic classification

Methods used by the NCCLS to assess cytogenetic abnormalities in leukemic cells have been previously described (Aldrich et al. 2006). Briefly, hospitals released medical record abstracts of participating cases that contained conventional banding karyotypes and occasional fluorescence *in situ* hybridization (FISH) screening. We conducted additional FISH analyses for ambiguous cases. We classified diagnostic karyotypes according to clonal genetic aberration using the International System for Human Cytogenetic Nomenclature 1995 criteria (Mitelman 1995). To date, cytogenetic information is available for 76% of ALL and 83% of AML cases included in the present study. We conducted analyses for major subgroups: for ALL, translocation between chromosomes 12 and 21 [t(12;21)], chromosomal deletions, hypodiploidy, and hyperploidy including low hyperdiploidy (47–50 chromosomes), high hyperdiploidy (51–68 chromosomes), and triploidy (69 chromosomes); for AML, we conducted analyses separately for the more favorable prognosis group, which encompasses any translocation involving chromosome 21, between chromosomes 15 and 17 t(15;17), and between chromosomes 8 and 16 t(8;16) and inversion on chromosome 16 [inv(16)], and for cases with any numerical change (Deschler and Lübbert 2006).

### Exposure assessment

We assessed household exposures to paint and petroleum solvents through an in-home personal interview conducted with the child’s biological mother (98% of interviews) or father (2%), which in addition to exposure to paint and petroleum solvents also included other chemical and pesticide exposures at home, occupational history, residential history, smoking habits, mother’s pregnancy and child’s delivery, mother’s reproductive history, child’s health/ medical history, family illness, and mother’s and child’s diets. We asked parents whether “paints, stains, or lacquers” (which we refer to as paint) and “adhesives or petroleum products, such as paint thinner, spot remover, paint remover, glue, solvent, gasoline, kerosene, or lubricating oil” (which we refer to as solvents) were ever used in their house within seven time windows (3 months before the pregnancy, during the first, the second, or the third trimester of the pregnancy, and from birth to 1 year of age, from 1 to 2 years of age, or from 2 to 3 years of age). We defined the reference date as the date of diagnosis for cases and corresponding date for matched controls. For children recruited until 2002, we censored exposures at the time window preceding the reference date (e.g., from birth to 1 year if the case was diagnosed between 1 and 2 years of age). Censoring exposures at the preceding time window was no longer necessary from 2003 onward because we changed the questionnaire to avoid possible recording of exposures that occurred after the reference date. We further detailed exposures to paint and solvents by user (mother, father, others) and frequency of use (fewer than five times, or five times or more) in the corresponding time window.

### Exposure prevalence and patterns

Overall, paint and petroleum solvents were used in households of 56% and 42% of cases, respectively, and 50% and 35% of control households. Table 2 further describes exposure patterns, by person who used the product, and by period when the product was used. For ALL or AML cases and controls and for both paint and solvent exposures, the father was the most frequent user and use was less common during pregnancy than after birth. Generally, households reported using paint less often than solvents in terms of median number of time windows when the product was used (one for paint vs. four for solvents) and frequency of use within each time window (25% had frequent use for paint vs. 47% for solvents).

### Statistical analyses

We conducted analyses separately for ALL and AML and by major cytogenetic subtypes, because evidence is increasing that these subtypes may differ in terms of risk factors. We calculated Spearman’s rank correlation coefficients between various time windows of exposure and highly correlated variables—that is, correlation coefficients > 0.80 were combined into one variable; this was the case only for use of solvent during the preconception and pregnancy periods combined into prenatal period. We used conditional logistic regression models to estimate the odds ratios (ORs) associated with home use of paint and solvents. These models employed binary variables for use of paint and/or solvents during the period of interest plus a series of potential confounders (household annual income, mother’s education level, father’s education level, breast-feeding, parental smoking, parental occupational exposure to paint and/or solvents, and parents’ age at child’s birth). We assessed the influences of these variables with likelihood ratio chi-square tests. Only income (ordinal variable) and father’s education level significantly improved the likelihood of the models (data not shown). However, because including the father’s education level in the models modified the estimates of the OR by < 2% (compared with ≥ 10% for income), we present only results adjusted for income. To investigate the role of the time window when the product was used, we included binary variables coding for use during preconception, during pregnancy, and postnatally in the model together and used likelihood methods to test their statistical significance. We tested whether the user (mother, father, others) affected the model’s likelihood using the same method. We tested interaction between matching factors [i.e., sex, age at diagnosis (reference) date, Hispanic status, and mother’s race] and paint and solvent exposure by including the interaction term in the regression models. For this purpose, we chose age categories (0–1.9, 2–5.9, 6–14.9 years of age) to fit the age-specific incidence curve of leukemia in childhood.

We created two categorical quantitative indexes for frequency of use separately for paint and solvents, taking into account the number of time windows when the product was used (median served as the threshold between categories) and the frequency during each time window: We considered use of paint rare if used only during one time window and fewer than five times, and frequent otherwise (i.e., more than five times in one time window or in two or more time windows); we considered use of solvents rare if used in four or fewer time windows and fewer than five times in each time window, and frequent otherwise (i.e., more than five times in one time window or in five or more time windows).

We calculated *p*-values for linear trend (*p*_trend_) when risk estimates showed a pattern consistent with a linear dose–response relationship. All statistical tests were two sided with α = 0.05.

## Results

Exposures to paint and petroleum solvents were not independent from each other [Spearman correlation coefficient (*r*) = 0.39, *p* < 0.001]. Proportions of households that used paint only, petroleum solvents only, and both were 23%, 9%, and 29%, respectively. The correlation array of detailed exposures by time windows (preconception, pregnancy, postnatal) showed low to moderate correlations (*r* ranged from 0.16 to 0.64), except for petroleum solvent exposure during preconception period and pregnancy (*r* = 0.80, *p* < 0.001). We combined these exposures into one “prenatal exposure” variable for the subsequent analyses. Household annual income was positively correlated with use of paint (*r* = 0.22, *p* < 0.001) and solvents (*r* = 0.23, *p* < 0.001).

### ALL

In univariate analyses, ORs were 1.49 [95% confidence interval (CI), 1.18–1.89] for paint exposure and 1.24 (95% CI, 0.97–1.59) for solvent exposure. After adjustment for income, they were 1.73 (95% CI, 1.35–2.21) and 1.39 (95% CI, 1.08–1.79), respectively. When we included both exposures in the model, OR for paint was 1.65 (95% CI, 1.26–2.15) and OR for solvent was 1.15 (95% CI, 0.87–1.51). We detected no significant interaction between the two exposures (*p* = 0.187). When we calculated risk estimates with a mutually exclusive categorical variable using nonusers of paint or solvents as the reference category, paint users had an OR of 1.84 (95% CI, 1.34–2.53), solvent users had an OR of 1.47 (95% CI, 0.93–2.32), and both exposures together produced an OR of 1.86 (95% CI, 1.37–2.53). We found no differences in ORs by child’s Hispanic status or sex or mother’s race, whereas the risk of childhood ALL somewhat varied by age category for exposure to solvents (*p* for heterogeneity = 0.077) but not paint (*p* = 0.359). Table 3 shows results for various users and time windows of exposures, stratified by age at diagnosis. Overall, the increased risk associated with paint exposure was statistically significant in the two youngest age categories (0- to 1.9-year-olds: OR = 2.70; 95% CI, 1.14–6.42; 2.0- to 5.9-year-olds: OR = 1.72; 95% CI, 1.21–2.45) but not in children diagnosed at ≥ 6 years of age (OR = 1.36; 95% CI, 0.84–2.20). We observed the increased risk associated with paint exposure when used by the father or others after birth. This observation was also true in the 2- to 5.9-year age category. On the contrary, ORs in the 0- to 1.9-year age category were higher when paint was used by the mother and before birth, although this finding was not statistically significant. There was a significant increased risk of ALL associated with solvent exposure in the 2- to 5.9-year age category (OR = 1.55; 95% CI, 1.07–2.25), but not in younger (OR = 0.66; 95% CI, 0.30–1.47) or in older children (OR = 0.87; 95% CI, 0.54–1.42).

Children of frequent users of paint had higher risk of ALL (OR = 1.74; 95% CI, 1.25–2.43) than did rare users (OR = 1.28; 95% CI, 0.92–1.78), with a statistically significant linear trend (*p* = 0.001). We found this dose–response relationship in both the 0- to 1.9-year age category (*p* = 0.031) and the 2.0- to 5.9-year age category (*p* = 0.010).

When the analyses were restricted to specific cytogenetic subtypes, paint exposure was associated with a significant increased risk of ALL with chromosomal structural changes, specifically with t(12;21) (Table 4). Among cases with t(12;21), ORs for preconception, pregnancy, and postnatal exposure to paint were 0.43 (95% CI, 0.11–1.64), 3.28 (95% CI, 1.12–9.60), and 4.15 (95% CI, 1.61–10.7), respectively. Concerning ALL cases with numerical chromosome changes in leukemic cells, the association with paint exposure was of borderline statistical significance (*p* = 0.056). We found no statistical significant results in relation to solvent exposure.

### AML

In univariate analyses, ORs were 0.83 (95% CI, 0.48–1.45) for paint exposure and 1.89 (95% CI, 1.00–3.49) for solvent exposure. After adjustment for income, the estimated ORs were 0.91 (95% CI, 0.51–1.63) and 2.05 (95% CI, 1.05–4.01), respectively. When we included both exposures in the model, the OR for paint was 0.64 (95% CI, 0.32–1.25) and for solvents was 2.54 (95% CI, 1.19–5.42) (Table 5). We detected no significant interaction between the two exposures (*p* = 0.270). We found no differences in risk estimates by Hispanic status, sex, age at diagnosis, or mother’s race. Table 5 presents detailed results by time window of use, type of user, and frequency. When analyzing the frequency of solvent use, the OR among rare users (*n* = 15) was higher than among frequent users (*n* = 35). Although based on small numbers, analyses by main cytogenetic subgroup (Table 6) showed elevated nonsignificant ORs for solvent exposure and AML cases with any structural change (OR = 2.50; 95% CI, 0.96–6.54), more specifically in the favorable prognosis group (OR = 3.49; 95% CI, 0.92–13.2), whereas we saw no association for AML with no detectable chromosome abnormalities.

## Discussion

In a large population-based case–control study that collected detailed self-reported home use of paint and solvents, our analyses identified a statistically significant association between paint exposure and ALL risk in childhood (OR = 1.65; 95% CI, 1.26–2.15), which was stronger in frequent users (OR = 1.74; 95% CI, 1.25–2.43) compared with rare users (OR = 1.28; 95% CI, 0.92–1.78) (*p*_trend_ = 0.001). Associations between ALL risk and solvent exposures were less consistent. In contrast, AML risk was significantly associated with solvent exposure (OR = 2.54; 95% CI, 1.19–5.42) but not with paint use (OR = 0.64; 95% CI, 0.32–1.25).

To our knowledge, no previous study has reported results of risks associated with paint and solvent exposures by cytogenetic subgroup of childhood leukemia. Yet this form of analysis may provide mechanistic explanations of the epidemiologic findings (Wiemels 2008). Although we observed no difference in risk estimates for the association between solvent exposure and ALL risk, our analyses have shown that association between paint exposure and ALL risk was statistically significant for cases with chromosomal structural changes in leukemic cells (OR = 1.96; 95% CI, 1.24–3.10) but of borderline significance for those with numerical changes (OR = 1.48; 95% CI, 0.99–2.21). Paint exposures appeared to be specifically related to ALL with t(12;21), but we are not sure whether they influence the identified genetic “hits” or influence the progression of the disease via other means, for instance, epigenetic changes or nongeno-toxic bone marrow suppression followed by regrowth (Wiemels et al. 2008). Indeed, our results showed that both pre- and postnatal exposures to paints conferred increased risk of ALL with t(12;21), hence supporting that timing of cell damages is unlikely to occur only *in utero*. Despite recent advances in the understanding of biological mechanisms leading to leukemia development, it is not known what factors might cause translocations to occur *in utero* (McHale and Smith 2004). How environmental exposures may modulate the progression of the preleukemic clones to leukemia is also unknown. The specificity of the association with paint toward t(12;21) over other subtypes, however, indicates a particular sensitivity of this leukemia to compounds contained in paints and deserves further research, with greater attention to exposure assessment by obtaining paint formulations from paint samples or manufacturers.

The possible influence of recall bias on the study results is probably the most important concern because differential recall between cases and controls can overestimate (or underestimate) an association in case–control design. Cases (or case parents) may have a tendency to better recall past exposures than do controls in an effort to elucidate what caused the disease their child has experienced. Although recall bias cannot be totally excluded, to minimize the use of open-ended questions and reduce the potential for recall bias, our questionnaire listed a range of products that we subsequently grouped into paint and petroleum solvent exposures, and parents were shown cards that illustrated the potentially exposed time windows. Also, if recall bias were to entirely explain our results, we would expect similar associations for ALL and AML; instead, we found distinct results (ALL being associated with paint, whereas AML was associated with solvent exposures). Participating controls who might not be representative of the general population where cases occurred is another important issue that might bias an association (selection bias). In our study, for example, participating controls had a higher annual income than did cases; socioeconomic status has been associated with the risk of childhood leukemia (Poole et al. 2006), and income was associated in our study with the use of paint and solvent at home. However, we adjusted all results for income and, if any effect remained, the higher proportion of high-income households among controls would have underestimated the risk estimates. Moreover, earlier analyses within our research group comparing several control groups demonstrated that the potential for selection bias in our study was minimal (Ma et al. 2004).

We evaluated potential confounders, such as parental smoking, breast-feeding, and parents’ ages, education level, and occupational exposure to paint and solvents, and none significantly influenced the risk estimates. We minimized confounding influences due to sex, age, and ethnicity by the matched-pair design. Because we individually matched the cases and controls, we maintained this control of potential confounders in the analysis by leukemia subtype. However, this approach cannot totally rule out potential confounding issues: Some confounders may have not been measured or may be too highly correlated with the exposure of interest. In our analyses of AML, adjusting solvent results for paint led to a higher OR (2.54 vs. 2.05 without adjustment) and adjusting paint results for solvents decreased the OR already below the unity (0.64 vs. 0.91). This might reflect differences in type of products used (e.g., if paint was used whereas no solvent was used, paint was likely to be water based) and could lead to spurious results after adjustment for the other exposure, especially when analyses are based on small numbers. The statistically significant lower risk of AML associated with use of paint by the father (after adjusting for income and solvent) is particularly difficult to interpret because we would expect no association or an increased risk. However, adjusting for income only led to a nonsignificant OR of 0.63 (95% CI, 0.30–1.32).

Lowengart et al. (1987) reported a statistically significant association between childhood leukemia (all types) and maternal use of paint during pregnancy (OR = 1.8, *p* = 0.03) based on 42 discordant case–control pairs, but attributed their results to chance because frequent users exhibited a lower and nonsignificant OR (OR = 1.3, *p* = 0.30, based on seven discordant pairs). However, Freedman et al. (2001) reported a significant dose–response relationship between the risk of ALL and the number of rooms painted in the house in the year preceding and after the child’s birth. When stratifying the analyses by time window of exposure, they observed a significant association only when paint was used after the child’s birth. We observed an association with the risk of ALL both during pregnancy and postnatally and further detailed the association by type of user. Analyzing all age groups together, the increased risk was statistically significant when paint was used by the father (OR = 1.44; 95% CI, 1.08–1.91) and by persons other than the parents (OR = 1.63; 95% CI, 1.17–2.26). The type of user may be correlated with quantity of paint used. Persons other than father and mother using paint in the house were likely to be professional painters or relatives who would help repaint rooms. In addition, parents might have used paint more frequently for hobbies in decreased quantities. Our data showed that the time window of exposure had some influence on the risk estimate, which was statistically significant when paint had been used after the child’s birth (OR = 1.39; 95% CI, 1.07–1.81) but not during pregnancy or in the preconception period. However, when examining the results in the 0- to 1.9-year age group, the OR was higher when paint had been used by the mother (OR = 2.49; 95% CI, 0.92–6.78) during preconception (OR = 2.91; 95% CI, 0.48–17.6) or pregnancy (OR = 1.92; 95% CI, 0.69–5.39). In the 2.0- to 5.9-year age group (which consists mainly of common ALL cases), results were comparable with the overall results, whereas in the older age group (≥ 6 years), risk estimates were closer to unity and not statistically significant. In the two younger age groups, ORs were higher for frequent users compared with rare users of paint (*p*_trend_ = 0.031 and 0.010 in the 0- to 1.9-year and 2.0- to 5.9-year age groups, respectively). Although these subgroup analyses suffer from low statistical power, they tend to show that time since exposure has a higher impact on risk estimates than specific time window of exposure or type of user. Our results suggest that paint exposure would have a rather proximate effect on the risk of ALL in offspring. This hypothesis would explain the lack of association in the 6.0- to 14.9-year age group, because we measured exposures only until age 3. On the other hand, leukemia occurring later in childhood has distinct clinical characteristics and possibly a different etiology. Adolescent ALL cases start to resemble adult ALL cases and do not usually have t(12;21); the lack of association between paint exposure and older ALL cases would then be consistent with the specificity toward cases with t(12;21). However, because reported paint exposures between birth and 3 years of age happened longer ago than for younger children, misclassification bias might be more important in older children and might partly explain the lack of association in this age group.

We detected the association between ALL risk and solvent exposure only in the 2.0- to 5.9-year age group after adjustment for paint (OR = 1.55; 95% CI, 1.07–2.25). We found no difference in risk estimates between time windows of use or users. False-positive results or unmeasured confounding factors cannot be excluded as possible explanations of the observed association with solvents in the 2.0- to 5.9-year age group. We detected no dose–response relationship, although frequency of use was more difficult to categorize than for paint because > 50% of households in our population that used solvents had used them in at least four time windows, and this regular use made classifications of “rare” and “frequent” users somewhat arbitrary. Also, the category of petroleum solvents consisted of a very broad and heterogeneous group of products, and errors in exposure classification might have biased risk estimates toward unity.

Because of the striking differences in the ORs for ALL risk between exposure to paint and to solvents, it is unlikely that the association observed with paint exposure is attributable to solvents contained in the paints. Household use of paint mainly involves latex paints, which typically contain vinyl acetate resin (polyvinyl acetate) or styrene-based resin (Kirk-Othmer 2006). Epidemiologic data and animal studies on polyvinyl acetate are too limited to allow evaluation of its potential carcinogenic risks in human beings, although further studies on this compound would be warranted when one considers that it has substantial commercial application (IARC Monograph Working Group 1979). On the other hand, styrene has been classified as possibly carcinogenic to human, and several studies have reported increased risks of lymphatic and hematopoietic neoplasms in workers exposed to this product (IARC Monograph Working Group 2002), which makes styrene a putative candidate to explain the association between paint exposure and ALL risk.

The association found between AML risk and petroleum solvent exposure was less consistent than between ALL risk and paint exposure. Obviously, the lower numbers of subjects in the AML group make these analyses more prone to false-positive and false-negative results. The overall risk estimate was large (OR = 2.54; 95% CI, 1.19–5.42), but we observed no dose-dependent pattern, with higher but unstable risk estimate in “rare” users than in “frequent” users (OR = 4.24; 95% CI, 1.24–14.5). However, our index of quantity of exposure might not be well suited to our population who reported using solvents regularly. The measurement of petroleum solvent exposures would benefit from more detail in the type of products used, and the resulting measurement error may have diluted our results. On the other hand, the analyses by cytogenetic subtype showed a higher OR in the favorable prognosis group of AML (OR = 3.49; 95% CI, 0.92–13.2), which is consistent with the chromosome banding patterns found in bone marrow cells of adult cases of AML that were exposed to solvents (Mitelman et al. 1981; Smith et al. 1998). Interestingly, one previous study focusing on childhood AML and its association with solvent exposure found a statistically significant association with paternal occupation exposure to petroleum solvents (Buckley et al. 1989).

In conclusion, our study corroborates earlier findings on the association between paint exposure at home and ALL risk in childhood and provides further evidence supportive of a causal relationship, such as the restriction of the association to cases with specific cytogenetic features and a higher OR among more frequent users. Although paint may consist of various compositions, unless specific causative agents are identified, avoiding the use of paint in the house during pregnancy and early childhood would be a prudent measure. An association between ALL risk and petroleum solvent exposure was suggested in the 2.0- to 5.9-year age group, although we observed no evidence of a dose–response relationship. AML risk was associated with petroleum solvent exposure, but smaller numbers complicated the interpretation of detailed analyses and more studies are needed to confirm these observations.

## Figures and Tables

**Table 1:** Selected characteristics of participating cases and controls [*n* (%)] by leukemia histological type: NCCLS.

	ALL		AML	
Characteristic	Cases	Controls	Cases	Controls
Total	550	737	100	125
Sex				
Male	311 (56.5)	415 (56.3)	55 (55.0)	68 (54.4)
Female	239 (43.5)	322 (43.7)	45 (45.0)	57 (45.6)
Age at reference date^a^ (years)				
0.0–1.9	62 (11.3)	80 (10.9)	30 (30.0)	37 (29.6)
2.0–5.9	313 (56.9)	425 (57.7)	20 (20.0)	28 (22.4)
6.0–14.9	175 (31.8)	232 (31.5)	50 (50.0)	60 (48.0)
Median (interquartile range)	4.5 (2.8–7.2)	4.4 (2.8–7.0)	6.1 (1.7–10.7)	5.8 (1.8–9.5)
Phase of the study				
Phase 1^b^	156 (28.4)	156 (21.2)	38 (38.0)	38 (30.4)
Phase 2^c^	394 (71.6)	581 (78.8)	62 (62.0)	87 (69.6)
Ethnicity				
Hispanic	247 (44.9)	320 (43.4)	39 (39.0)	49 (39.2)
Non-Hispanic white	221 (40.2)	303 (41.1)	40 (40.0)	55 (44.0)
Non-Hispanic other	82 (14.9)	114 (15.5)	21 (21.0)	21 (16.8)
Household income (US$ 1,000/year)				
< 15	87 (15.8)	76 (10.3)	21 (21.0)	11 (8.8)
15–29	98 (17.8)	105 (14.2)	17 (17.0)	17 (13.6)
30–44	84 (15.3)	95 (12.9)	16 (16.0)	15 (12.0)
45–59	89 (16.2)	100 (13.6)	10 (10.0)	20 (16.0)
60–74	48 (8.7)	80 (10.9)	12 (12.0)	14 (11.2)
≥ 75	144 (26.2)	281 (38.1)	24 (24.0)	48 (38.4)
Father’s education level				
None or elementary school	56 (10.2)	79 (10.7)	11 (11.0)	12 (9.6)
High school or similar	196 (35.6)	224 (30.4)	36 (36.0)	43 (34.4)
Some college or similar	116 (21.1)	188 (25.5)	18 (18.0)	29 (23.2)
Bachelors degree or higher	163 (29.6)	216 (29.3)	32 (32.0)	40 (32.0)
Missing	19 (3.5)	30 (4.1)	3 (3.0)	1 (0.8)
Mother’s education level				
None or elementary school	69 (12.5)	58 (7.9)	13 (13.0)	11 (8.8)
High school or similar	168 (30.5)	210 (28.5)	30 (30.0)	35 (28.0)
Some college or similar	159 (28.9)	239 (32.4)	24 (24.0)	33 (26.4)
Bachelors degree or higher	154 (28.0)	229 (31.1)	33 (33.0)	46 (36.8)
Missing	0 (0.0)	1 (0.1)	0 (0.0)	0 (0.0)
Father’s age at child’s birth (years)				
Median (interquartile range)	31.0 (25.7–35.6)	31.2 (26.5–35.8)	29.6 (25.5–34.9)	32.4 (26.0–35.5)
Mean ± SD	30.9 ± 7.1	31.4 ± 7.0	30.8 ± 7.2	31.5 ± 6.6
Mother’s age at child’s birth (years)				
Median (interquartile range)	28.8 (23.5–32.9)	28.8 (24.2–33.5)	27.7 (23.2–31.6)	29.6 (25.5–33.2)
Mean ± SD	28.4 ± 6.1	29.0 ± 6.1	27.9 ± 6.4	29.4 ± 6.0

a Reference date was defined as the date of diagnosis for cases and corresponding date for matched controls.

b August 1995 through November 1999, covering 17 counties: Alameda, Contra Costa, Marin, Merced, Monterey, Napa, Sacramento, San Benito, San Francisco, San Joaquin, San Mateo, Santa Clara, Santa Cruz, Solano, Sonoma, Stanislaus, and Yolo.

c December 1999 through December 2005, covering 35 counties: 17 counties from phase 1, plus Amador, Butte, Calaveras, Colusa, El Dorado, Fresno, Glenn, Kern, Kings, Madera, Mariposa, Nevada, Placer, San Luis Obispo, Sutter, Tulare, Tuolumne, and Yuba.

**Table 2:** Characteristics of paint and petroleum solvent use in households of children participating in the NCCLS [*n* (%)].

	ALL		AML	
User and time window of use	Cases (*n* = 550)	Controls (*n* = 737)	Cases (*n* = 100)	Controls (*n* = 125)
Paint				
Overall	322 (58.5)	366 (49.7)	44 (44.0)	61 (48.8)
By mother	172 (31.3)	206 (28.0)	24 (24.0)	32 (25.6)
By father	203 (36.9)	229 (31.1)	22 (22.0)	37 (29.6)
By another person	105 (19.1)	95 (12.9)	14 (14.0)	14 (11.2)
During the 3 months before pregnancy	54 (9.8)	61 (8.3)	12 (12.0)	9 (7.2)
During first trimester	51 (9.3)	46 (6.2)	11 (11.0)	8 (6.4)
During second trimester	53 (9.6)	54 (7.3)	12 (12.0)	10 (8.0)
During third trimester	84 (15.3)	95 (12.9)	16 (16.0)	17 (13.6)
From birth to first birthday/reference date^a^	115 (21.4)	134 (18.5)	19 (20.9)	21 (17.5)
From first birthday to second birthday/reference date^b^	126 (24.9)	151 (22.0)	20 (25.6)	22 (21.8)
From second birthday to third birthday/reference date^c^	120 (28.0)	153 (25.8)	16 (24.6)	20 (23.5)
Petroleum solvents				
Overall	230 (41.8)	270 (36.6)	40 (40.0)	35 (28.0)
By mother	102 (18.5)	128 (17.4)	16 (16.0)	15 (12.0)
By father	158 (28.7)	177 (24.0)	31 (31.0)	28 (22.4)
By another person	41 (7.5)	39 (5.3)	5 (5.0)	5 (4.0)
During the 3 months before pregnancy	113 (20.5)	132 (17.9)	17 (17.0)	21 (16.8)
During first trimester	110 (20.0)	135 (18.3)	17 (17.0)	21 (16.8)
During second trimester	112 (20.4)	142 (19.3)	19 (19.0)	22 (17.6)
During third trimester	120 (21.8)	144 (19.5)	21 (21.0)	21 (16.8)
From birth to first birthday/reference date^a^	139 (25.8)	172 (23.8)	20 (22.0)	24 (20.0)
From first birthday to second birthday/reference date^b^	144 (28.5)	170 (24.8)	22 (28.2)	24 (23.8)
From second birthday to third birthday/reference date^c^	132 (30.8)	155 (26.1)	19 (29.2)	20 (23.5)

a Denominators = 538 for ALL cases, 724 for ALL controls, 91 for AML cases, and 120 for AML controls.

b Denominators = 506 for ALL cases, 685 for ALL controls, 78 for AML cases, and 101 for AML controls.

c Denominators = 428 for ALL cases, 593 for ALL controls, 65 for AML cases, and 85 for AML controls.

**Table 3:** ORs (95% CIs) of ALL for household use of paint and solvents for various users and time windows of use, stratified by age at diagnosis (conditional logistic regressions, adjustment for income and either paint or solvent exposure).

			Age at diagnosis (years)					
	All cases (*n* = 550)		0–1.9 (*n* = 62)		2.0–5.9 (*n* = 313)		6.0–14.9 (*n* = 175)	
Exposure	No. of discordant pairs/triplets (%)	OR (95% CI)	No. of discordant pairs/triplets (%)	OR (95% CI)	No. of discordant pairs/triplets (%)	OR (95% CI)	No. of discordant pairs/triplets (%)	OR (95% CI)
Paint								
Overall	302 (54.9)	1.65 (1.26–2.15)	31 (50.0)	2.70 (1.14–6.42)	181 (57.8)	1.72 (1.21–2.45)	90 (51.4)	1.36 (0.84–2.20)
By period of exposure								
Preconception	106 (19.3)	1.10 (0.71–1.69)	10 (16.0)	2.91 (0.48–17.6)	55 (17.6)	1.04 (0.58–1.87)	41 (23.4)	1.08 (0.53–2.24)
Pregnancy	196 (35.6)	1.21 (0.88–1.67)	27 (43.5)	1.92 (0.69–5.39)	107 (34.2)	1.21 (0.78–1.88)	62 (35.4)	1.12 (0.62–2.02)
After birth	292 (53.1)	1.39 (1.07–1.81)	25 (40.3)	1.11 (0.45–2.76)	176 (56.2)	1.46 (1.13–2.38)	91 (52.0)	1.35 (0.84–2.17)
By user								
Mother	252 (45.8)	1.19 (0.89–1.58)	24 (38.7)	2.49 (0.92–6.78)	155 (49.5)	1.20 (0.83–1.75)	73 (41.7)	1.06 (0.61–1.84)
Father	281 (51.1)	1.44 (1.08–1.91)	27 (43.5)	2.06 (0.80–5.27)	170 (54.3)	1.50 (1.04–2.17)	84 (48.0)	1.29 (0.76–2.19)
Others	166 (30.2)	1.63 (1.17–2.26)	14 (22.6)	0.83 (0.24–2.84)	95 (30.6)	1.85 (1.17–2.93)	57 (32.6)	1.45 (0.85–2.49)
Frequency of use^a^								
Rarely	154 (28.0)	1.28 (0.92–1.78)	21 (33.9)	2.36 (0.89–6.29)	95 (30.4)	1.40 (0.90–2.17)	38 (21.7)	0.83 (0.44–1.59)
Frequently	194 (35.3)	1.74 (1.25–2.43)	15 (24.2)	3.50 (1.08–11.4)	115 (36.7)	1.77 (1.13–2.75)	64 (36.6)	1.48 (0.83–2.62)
*p*_trend_^b^		0.001		0.031		0.010		—
Solvents								
Overall	271 (49.3)	1.15 (0.87–1.51)	32 (51.6)	0.66 (0.30–1.47)	150 (47.9)	1.55 (1.07–2.25)	89 (50.9)	0.87 (0.54–1.42)
By period of exposure								
Before birth	214 (38.9)	1.19 (0.83–1.71)	28 (45.2)	1.49 (0.49–4.49)	121 (38.7)	1.20 (0.74–1.95)	65 (37.1)	1.13 (0.60–2.14)
After birth	245 (44.5)	1.06 (0.76–1.50)	21 (33.9)	0.60 (0.19–1.87)	145 (46.3)	1.26 (0.80–1.98)	79 (45.1)	0.93 (0.50–1.72)
By user								
Mother	161 (29.3)	1.08 (0.77–1.51)	17 (27.4)	1.00 (0.36–2.78)	101 (32.3)	1.41 (0.91–2.20)	43 (24.6)	0.70 (0.36–1.38)
Father	237 (43.1)	1.17 (0.88–1.56)	29 (46.8)	0.66 (0.27–1.57)	137 (43.8)	1.44 (0.98–2.11)	71 (40.6)	1.06 (0.63–1.78)
Others	79 (14.4)	1.16 (0.73–1.86)	8 (12.9)	2.66 (0.60–11.8)	40 (12.8)	1.10 (0.56–2.17)	31 (17.7)	1.15 (0.52–2.52)
Frequency of use^c^								
Rarely	109 (19.8)	1.12 (0.75–1.67)	14 (22.6)	0.42 (0.12–1.40)	62 (19.8)	1.78 (1.04–3.04)	33 (18.9)	0.59 (0.26–1.33)
Frequently	180 (32.7)	1.31 (0.95–1.81)	20 (32.3)	1.06 (0.41–2.75)	99 (31.6)	1.50 (0.96–2.34)	61 (34.9)	1.22 (0.70–2.13)
*p*_trend_^b^		0.102		—		—		—

a “Rarely” indicates one time window only (preconception, first, second, and third trimester of pregnancy, first, second, and third year of life) and fewer than five times; “Frequently” indicates other combinations.

b *p*-Value obtained when entered as a continuous variable in the model (calculated only when risk estimates showed a pattern consistent with a linear dose–response relationship).

c “Rarely” indicates four time windows or fewer (preconception, first, second, and third trimester of pregnancy, first, second, and third year of life), and fewer than five times in each; “Frequently” indicates other combinations.

**Table 4:** ORs (95% CIs) of ALL for household use of paint and solvents by cytogenetic subtype (conditional logistic regressions, adjustment for income, and either paint or solvent exposure).

		Paint exposure		Petroleum solvent exposure	
Cytogenetic subtype	No. of case–control sets	No. of discordant pairs/triplets (%)	OR (95% CI)	No. of discordant pairs/triplets (%)	OR (95% CI)
“Normal” karyotype	85	38 (44.7)	1.78 (0.87–3.64)	39 (45.9)	0.87 (0.43–1.76)
Abnormal karyotype	309	166 (53.7)	1.61 (1.13–2.30)	152 (49.2)	1.07 (0.74–1.53)
Any structural change	190	105 (55.3)	1.96 (1.24–3.10)	102 (53.7)	0.86 (0.54–1.34)
t(12;21)	67	38 (56.7)	4.16 (1.66–10.4)	39 (58.2)	0.98 (0.45–2.15)
Any deletion	45	21 (46.7)	0.98 (0.39–2.49)	22 (48.9)	0.74 (0.30–1.81)
Other structural change	83	47 (56.6)	1.63 (0.81–3.26)	43 (51.8)	0.84 (0.41–1.72)
Any numerical change	227	124 (54.6)	1.48 (0.99–2.21)	108 (47.6)	1.05 (0.69–1.61)
Hyperploidy (including triploidy)	186	103 (55.4)	1.42 (0.92–2.19)	85 (45.7)	1.18 (0.74–1.89)
Hypodiploidy	24	15 (62.5)	2.18 (0.47–9.99)	14 (58.3)	0.62 (0.13–2.87)

**Table 5:** ORs (95% CIs) of AML for household use of paint and solvents for various users and time windows of use (conditional logistic regressions, adjustment for income, and either paint or solvent exposure).

Exposure	No. of discordant pairs/triplets (%)	OR (95% CI)
Paint				
Overall	52 (52.0)	0.64 (0.32–1.25)
By period of exposure				
Preconception	20 (20.0)	1.35 (0.40–4.57)
Pregnancy	32 (32.0)	0.99 (0.37–2.62)
After birth	47 (47.0)	0.86 (0.41–1.77)
By user				
Mother	37 (37.0)	1.37 (0.61–3.11)
Father	43 (43.0)	0.39 (0.17–0.92)
Others	27 (27.0)	0.82 (0.33–2.06)
Frequency of use^a^				
Rarely	26 (26.0)	0.44 (0.18–1.07)
Frequently	33 (33.0)	1.37 (0.59–3.16)
Solvents				
Overall	44 (44.0)	2.54 (1.19–5.42)
By period of exposure				
Before birth	42 (42.0)	0.87 (0.33–2.32)
After birth	36 (36.0)	2.13 (0.77–5.86)
By user				
Mother	23 (23.0)	1.64 (0.58–4.62)
Father	37 (37.0)	1.94 (0.87–4.32)
Others	10 (10.0)	1.04 (0.23–4.64)
Frequency of use^b^				
Rarely	15 (15.0)	4.24 (1.24–14.5)
Frequently	35 (35.0)	2.23 (1.01–4.93)

a “Rarely” indicates one time window only (preconception, first, second, and third trimester of pregnancy, first, second, and third year of life) and fewer than five times; “Frequently” indicates other combinations.

b “Rarely” indicates four time windows or fewer (preconception, first, second, and third trimester of pregnancy, first, second, and third year of life), and fewer than five times in each; “Frequently” indicates other combinations.

**Table 6:** ORs (95% CIs) of AML for household use of paint and solvents by cytogenetic subtype (conditional logistic regressions, adjustment for income, and either paint or solvent exposure).

		Paint exposure		Petroleum solvent exposure	
Cytogenetic subtype	No. of case–control sets	No. of discordant pairs/triplets (%)	OR (95% CI)	No. of discordant pairs/triplets (%)	OR (95% CI)
“Normal” karyotype	16	10 (62.5)	0.25 (0.04–1.42)	9 (56.2)	1.05 (0.11–10.4)
Abnormal karyotype	65	30 (46.1)	0.86 (0.37–2.01)	28 (43.1)	2.12 (0.87–5.18)
Any structural change	59	26 (44.1)	0.96 (0.39–2.35)	24 (40.7)	2.50 (0.96–6.54)
Good prognosis group^a^	28	15 (53.6)	0.70 (0.22–2.27)	13 (46.4)	3.49 (0.92–13.2)
Other	31	11 (35.5)	2.00 (0.38–10.5)	11 (35.5)	1.09 (0.22–5.39)
Any numerical change	35	14 (40.0)	1.62 (0.43–6.18)	14 (40.0)	0.78 (0.21–2.85)

a Includes any translocation involving chromosome 21, t(15;17), t(8;16), and inv(16)
